# Altered expression of T cell Immunoglobulin-Mucin (TIM) molecules in bronchoalveolar lavage CD4^+ ^T cells in sarcoidosis

**DOI:** 10.1186/1465-9921-10-42

**Published:** 2009-05-29

**Authors:** Farah Idali, Jan Wahlström, Benita Dahlberg, Mohsen Khademi, Tomas Olsson, Anders Eklund, Johan Grunewald

**Affiliations:** 1 Department of Medicine, Unit of Respiratory Medicine, Karolinska Institutet, Stockholm, Sweden; 2 Monoclonal Antibody Research Center, Avicenna Research Institute, ACECR, Tehran, Iran; 3 Department of Clinical Neuroscience, Unit of Neuroimmunology, Karolinska Institutet, Stockholm, Sweden

## Abstract

**Background:**

Activated T helper (Th)-1 pulmonary CD4^+ ^cells and their mediators are essential for the inflammation and granulomatous process in sarcoidosis. Recently, T-cell immunoglobulin and mucin domain (TIM) molecules were suggested to be important regulators of immune function. In this study, we wanted to investigate whether TIM molecules could play a role in sarcoidosis.

**Methods:**

We used real-time polymerase chain reaction to investigate the differential gene expression of TIM-1 and TIM-3 as well as a few Th1 and Th2 cytokines (IL-2, IFN-γ, IL-4, IL-5 and IL-13) in CD4^+ ^T cells isolated from bronchoalveolar lavage fluid (BALF) of patients *(n = 28) *and healthy controls *(n = 8)*. Using flow cytometry, we were also able to analyse TIM-3 protein expression in 10 patients and 6 healthy controls.

**Results:**

A decreased TIM-3 mRNA *(p < 0.05) *and protein *(p < 0.05) *expression was observed in patients, and the level of TIM-3 mRNA correlated negatively with the CD4/CD8 T cell ratio in BALF cells of patients. Compared to a distinct subgroup of patients i.e. those with Löfgren's syndrome, BALF CD4^+ ^T cells from non- Löfgren's patients expressed decreased mRNA levels of TIM-1 *(p < 0.05)*. mRNA expression of IL-2 was increased in patients *(p < 0.01) *and non-Löfgren's patients expressed significantly higher levels of IFN-γ mRNA *(p < 0.05*) versus patients with Löfgren's syndrome.

**Conclusion:**

These findings are the first data on the expression of TIM-1 and TIM-3 molecules in sarcoidosis. The reduced TIM-3 expression in the lungs of patients may result in a defective T cell ability to control the Th1 immune response and could thus contribute to the pathogenesis of sarcoidosis. The down-regulated TIM-1 expression in non-Löfgren'spatients is in agreement with an exaggerated Th1 response in these patients.

## Background

Sarcoidosis is a T helper (Th) 1-mediated inflammatory disease with unknown aetiology, characterized by the formation of noncaseating granulomas, and involving accumulations of macrophages and T cells, primarily affecting the lungs [1]. In pulmonary sarcoidosis, an acute onset usually indicates a self-limiting disease course, whereas an insidious onset may be followed by persistent disease with a risk for fibrosis [1]. An imbalance in the expression of Th1/Th2 cytokines by alveolar cells has been suggested to be of importance for the outcome of a pulmonary immune response in sarcoidosis [2-4]. Löfgren's syndrome is regarded as a distinct clinical group with an acute onset of disease with erythema nodosum (EN) and/or ankle arthritis, bilateral hilar lymphadenopathy (BHL), fever and with a characteristic favourable disease outcome, often with complete spontaneous resolution. In contrast, non-Löfgren's patients more often have an insidious onset of disease with symptoms such as dry cough, low grade fever, fatigue, shortness of breath and more pronounced chest radiographic changes.

The human leukocyte antigen (HLA)-DRB1*0301 allele has been reported to be overrepresented in sarcoidosis patients with Löfgren's syndrome [5]. Additional studies have revealed a strong association between HLA-DRB1*0301 and remarkable expansions of CD4^+ ^T cells expressing T-cell receptors using the AV2S3 gene segment in bronchoalveolar lavage fluid (BALF) of Scandinavian sarcoidosis patients [6,7]. The expansion of AV2S3^+ ^cells at disease onset was found to correlate with a better prognosis, suggesting a protective role for these cells in sarcoidosis [8]. However, any functional role of these cells has not been determined.

Recent investigations of mechanisms that regulate activation and function of CD4^+ ^T cells have shown that T cell immunoglobulin -mucin (TIM) proteins are important regulators of immune function. TIM family members are type I transmembrane proteins, with extracellular immunoglobulin and mucin domains and intracellular domains of different lengths. TIMs are differentially expressed on Th1 and Th2 cells [9,10]. The TIM gene family includes eight genes in mice and three genes in human. In humans, the gene encoding the TIM-1 protein has been considered as an important atopy susceptibility gene and is associated with Th2 T cell responses [11], suggesting that TIM-1 controls critical regulatory pathways in the immune system. Studies on mice have indicated that TIM-1 is involved in T helper cell differentiation and suggested that the protein is a positive regulator of T cell activity [12,13]. Furthermore, blocking of TIM-1 during *in vivo *development of T-cell responses in a mouse model of asthma was followed by decreased Th2 immune responses and airway inflammation [14]. Another member of TIM family proteins is TIM-3, which in contrast to TIM-1 preferentially is expressed on fully differentiated CD4^+ ^Th1 cells but not on Th2 cells [9,15]. The ligand for TIM-3 has been identified as galectin-9, with expression mostly on CD4^+ ^T cells [16]. Galectin-9 has been shown to induce apoptosis of T cells via the calcium-calpain- caspase-1 pathway [17]. In mice, interference with the TIM-3-TIM-3-ligand binding resulted in hyperproliferation of Th1 cells with spontaneous production of Th1 cytokines [18], as well as macrophage activation and accelerated Th1-mediated autoimmunity [19]. Accumulating data suggest that TIM-3 negatively regulates Th1-type immune responses [16,18].

In the current study we examined the mRNA expression of TIM-1 and TIM-3 as well as Th1 and Th2 cytokines in CD4^+ ^T cells sorted by means of flow cytometry from BALF of patients with active sarcoidosis and healthy subjects. In addition using flow cytometry, the protein expression of TIMs on BALF and blood Tcells was investigated. The patients were stratified depending on whether they had Löfgren's syndrome or not. The expression of the same genes was also investigated in compartmentalized BALF CD4^+^AV2S3^+ ^T cells from patients with lung restricted AV2S3 T cell expansions.

## Methods

### Study subjects

The study was performed on 47 patients with pulmonary sarcoidosis and 14 healthy controls (table 1). All patients, including both Löfgren's and non-Löfgren's patients, had an active, symtomatic disease and were consecutively included as they were referred for diagnostic purposes for the first time to the Division of Respiratory Medicine, Karolinska University Hospital, Stockholm, Sweden. Patients had a clinical picture in accordance with pulmonary sarcoidosis, as determined by symptoms (such as cough, shortness of breath and fatigue), chest radiography and pulmonary function tests, and the diagnosis was established using the criteria by the World Association of Sarcoidosis and other Granulomatous disorders (WASOG) [1]. No patient was on treatment with immunosuppressive drugs.

**Table 1:** BALF analysis and lung function parameters

	Löfgren's (n = 26)	Non-Löfgren's (n = 21)	Controls (n = 14)
Sex, male/female	12/14	9/12	5/9
Age, yr	39 (25–59)**†	50 (34–75)***	28 (21–39)
X-ray stage (0/I/II/III)	0/17/8/1	0/5/9/4 (3 ND)	14/0/0/0
			
BAL analyses			
% recovery	72 (44–82)	67 (44–80)	72 (61–85)
% viability	95 (85–99.6)	95 (82–98.2)	95 (86–98)
Cell concentration (*10^6^/L)	196 (49–588)**	219 (84–746)***	101 (50–167)
			
Differential cell counts			
% macrophages	72 (39.4–91)**	62.2 (37–91)***	87 (65–95)
% lymphocytes	28 (7.6–58)**	34.6 (8.2–61)***	11 (3.8–29)
% neutrophils	0.9 (0–4.8)	1.0 (0–6.0)	1.6 (0.2–4.4)
% eosinophils	0.2 (0–1.8)	0.5 (0.–4.4)	0.5 (0–1.6)
			
CD4/CD8 ratio	8.7 (2.4–28.4)	7.2 (0.9–46)	ND
HLA-DRB1*0301	20 of 24 (2 ND)	2 of 19 (2 ND)	ND
AV2S3 expansion^1)^	21 of 26	4 of 19 (2 ND)	ND
			
Pulmonary function tests			
VC (% of ref value)	90 (68–126)	78 (53–133)	ND
FEV1 (% of ref value)	88 (66–122)†	74 (56–131)	ND
DLco (% of ref value)	83 (66–126)	81 (54–106)	ND

Data are shown as median (min-max)

VC: Vital Capacity

FEV_1_: Forced Expiratory Volume in the first second

DLco: Diffusing capacity of the lung for carbon monoxide

**p < 0.01 versus healthy controls

*** p < 0.001 versus healthy controls

† p < 0.05 between patient subgroups

ND, not done

1) Defined as at least three times higher than the corresponding median value in PBMCs from healthy controls i.e. ≥ 10.5%.

Patients were divided into two groups; those with Löfgren's syndrome *(n = 26)*, and those without *(n = 21)*. BALF CD4^+ ^T cells were isolated from 28 of the patients; 13 with Löfgren's syndrome and 15 non-Löfgren's patients. FACS sorted BALF CD4^+ ^T cells from 8 non-smoking healthy adults were included as controls.

In addition, from 12 of the patients with lung restricted expansions of AV2S3^+ ^T cells (≥ 10.5% of CD4^+ ^cells in BALF) [6,20], we isolated BALF CD4^+ ^T cells either expressing the TCR AV2S3 gene segment (CD4^+^AV2S3^+^) or not (CD4^+ ^AV2S3^-^). All except one had Löfgren's syndrome. In order to obtain a sufficient number of CD4^+^AV2S3^+ ^T cells, in all but three patients we had to choose to sort either CD4^+ ^T cells or CD4^+^AV2S3^+ ^T cells.

Finally, we analysed by flow cytometry TIM-1 and TIM-3 proteins on BALF and blood T cells of 10 patients (four with Löfgren's syndrome) and 6 healthy subjects, using antibodies that were made commercially available during the course of the study.

All subjects gave their informed consent to participate in the study, and the local ethics committee, the Regional Ethical Review Board in Stockholm (ref. nr: 2005/1031-31) approved the study.

### Bronchoalveolar lavage (BAL)

Bronchoalveolar lavage was performed as described [21]. Briefly, fibreoptic bronchoscopy was performed under local anaesthesia on patients. A flexible fiberoptic bronchoscope (OBF Type 1 TR; Olympus Optical Co., Japan) was passed transorally and wedged into the middle-lobe bronchus and sterile phosphate-buffered saline (PBS) solution at 37°C was instilled in five aliquots of 50 ml and immediately re-aspirated and collected in a siliconized plastic bottle that was kept on ice.

The BAL fluids recovered were centrifugated at 400 g for 10 min at 4°C, to separate BAL cells from the supernatant. The cell pellet was resuspended in RPMI-1640 medium (Sigma-Aldrich, Irvin, UK) and the viability was determined by trypan blue exclusion. Cell differential counts were determined by May-Grünwald-Giemsa staining of cytopin slides.

### Flow cytometric analysis and isolation of cells

BALF CD4/CD8 T lymphocyte ratio and TCR AV2S3 expression in BALF cells was determined by FACS analysis using monoclonal antibodies (Mabs) against CD3^+^, CD4^+ ^and CD8^+ ^(Dako Cytomation Norden AB, Solna, Sweden) and anti-human TCR AV2S3 (clone F1) (Pierce Biotechnology, Rockford, USA), as previously described [22].

For sorting, cells were stained with anti- CD4-Phycoerythrin (PE) (DAKO) and anti-TCR AV2S3-Flourescein isothiocyanate (FITC) (Pierce Biotechnology). The stained cells were sorted by FACSVantage (BD Biosciences, Montain View, California, USA). BALF cells were gated on lymphocytes and sorted into different populations; CD4^+ ^T cells from patients and controls, as well as CD4^+ ^AV2S3^+ ^and CD4^+ ^AV2S3^- ^T cells from patients with lung accumulated T cells expressing the AV2S3 TCR gene segment. The purity of the sorted populations, which was determined by FACS, was 98% on average.

The analysis of TIM-1 and TIM-3 cell surface expression in blood and BALF CD4^+ ^and CD8^+ ^T cells was performed on BD FACSCanto II flow cytometer with FACSDiva software (BD Biosience). 1 × 10^6 ^BALF cells or 100 μl heparinized blood were surface stained with primary monoclonal antibody against human TIM-1 (R&D system, Minneapolis, Minnesota, USA), followed by APC- conjugated goat anti-mouse antibody. Cells were washed and blocked with normal mouse serum. Subsequently, cells were incubated for 20 minutes with anti-human CD3-pacific blue (BD Biosciences), CD4-APC H7 (BD Biosciences), CD8-PE-cy5 (BD Biosciences), AV2S3-FITC (Pierce Biotechnology) and TIM-3-PE (R&D system) monoclonal antibodies. Mouse IgG1-FITC (BD Biosciences), mouse IgG2b (Nordic Biosite AB, Stockholm, Sweden) and rat IgG2a (BD Biosciences) were used as isotype controls. The red blood cells were lysed after the end of incubations. Expression of cell surface markers was determined by flow cytometry after gating on either CD3^+^CD4^+^or CD3^+^CD8^+ ^lymphocytes.

### Quantitative analysis of the gene expression by real-time polymerase chain reaction (PCR)

Total RNA was extracted and cDNA was synthesized. Gene expression was quantified by real-time PCR using ABI Prism 7700 Sequence Detection System (Applied Biosystems, Foster City, CA, USA), as described [23]. RNA specimens were analyzed in duplicate using primers and probes for β-actin [23], TIM-1 and total TIM-3 [15] as well as the Assay-On-Demand products for IFN-γ (Hs00174143_m1), IL-2 (Hs00174114_m1), IL-13 (Hs00174379_m1), IL-4 (Hs00929861_g1), IL-5 (Hs99999031-m1) and galectin-9 (Hs00247135-m1). The Assay-On-Demand products and universal master mix were commercially purchased (Applied Biosystems). All samples were run in duplicates and the mean values calculated.

For relative quantification of expression of each gene in BALF cells, the following arithmetic formula was used: 2^-ΔΔCT ^(Perkin-Elmer Instruction manual, 1997), where the amount of target gene was normalized to β-actin (house-keeping gene) and the relative expression of a gene in BALF was calculated in relation to the mean value of target gene expression in the healthy control group.

### Human leukocyte antigen typing

HLA class II (HLA-DR) typing was done on DNA by PCR amplification using sequence specific primers [24].

### Statistics

Significance levels were calculated according to nonparametric tests, using Mann-Whitney U test for comparison between two groups or the Kruskal-Wallis test followed by Dunn's post-test for comparisons between groups. The non-parametric Wilcoxon matched pairs statistical test was used for calculation of statistical significances of TIM-3 expression between BAL and blood in patients and controls. Correlations between different parameters were determined with Spearman's rank correlation test. Values of *p < 0.05 *were regarded as significant. All statistical analyses were performed with Graphpad Prism 4.03 (Graphpad software Inc, San Diego, California, USA).

## Results

### BALF analysis and lung function parameters

Table 1 shows the BALF cell characteristics and the results of pulmonary function tests in the patients and controls. Whereas BAL cell concentrations and percentages of lymphocytes in each patient subgroup compared to controls were increased, the percentages of macrophages were decreased in patients. There was also a tendency to increased BAL cell concentrations in non-Löfgren's patients versus Löfgren's patients. The forced expiratory volume in one second (FEV1) was lower in non-Löfgren's sarcoidosis patients than in Löfgren's patients.

### TIM mRNA expression in CD^4+ ^T cells

**Figure 1:**
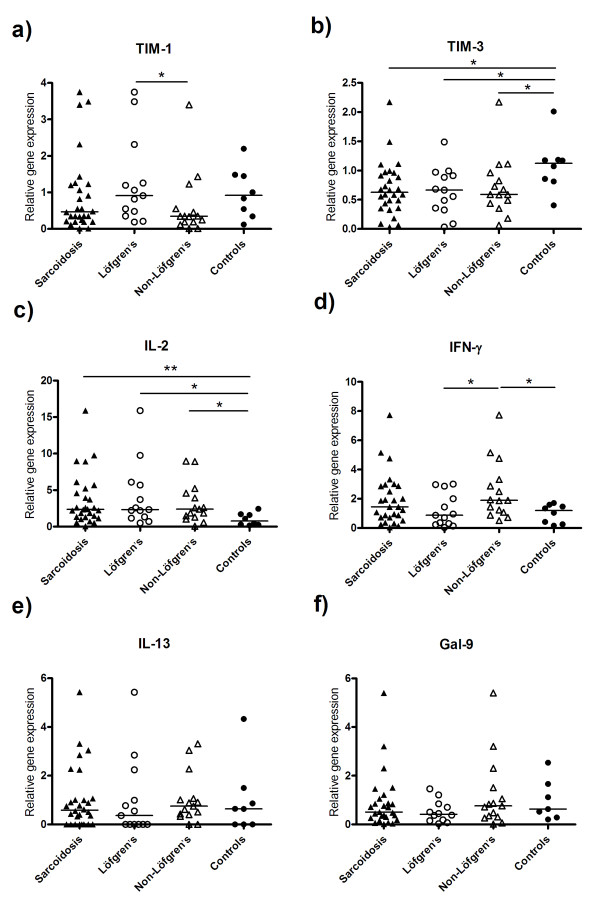
**The relative RNA transcript for a) TIM-1, b) TIM-3, c) IL-2, d) IFN-γ, e) IL-13 and f) galectin-9 (gal-9) in BALF CD4^+ ^T cells of all sarcoidosis patients *(n = 28)*, including patient subgroups: patients with Löfgren's syndrome *(n = 13)*, non-Löfgren's *(n = 15) *patients and healthy controls *(n = 8) *was measured**. TIM-3 mRNA levels were significantly decreased in sarcoidosis patients compared to controls *(p = 0.02)*, while the mRNA levels of IL-2 were significantly increased in sarcoidosis patients comparing to controls *(p = 0.008)*. The relative TIM-1 mRNA levels were significantly decreased *(p = 0.03)*, while IFN-γ mRNA expression was elevated *(p = 0.03) *in non- Löfgren's patients compared to Löfgren's patients. Horizontal bars indicate median values. * p < 0.05, ** p < 0.01.

The mRNA expression of TIMs was investigated in FACS-sorted BALF CD4^+ ^T cells. Using real time PCR, we found no significant differences in the mRNA levels of TIM-1 between patients and controls (figure 1a). However, analysing the gene expression in patient subgroups showed that there was a significant decrease in TIM-1 expression in non-Löfgren's patients *(n = 15) *versus patients with Löfgren's syndrome *(n = 13) *(*p = 0.03*; figure 1a). A significant reduction in TIM-3 mRNA expression was observed in patients compared to controls (*p = 0.02*; figure 1b). There was no difference in TIM-3 mRNA expression between Löfgren's and non-Löfgren's patients, but both subgroups showed significantly decreased levels of TIM-3 compared to controls (figure 1b).

### Cytokine mRNA expression in CD^4+ ^T cells

We also analyzed the expression of Th1 and Th2 cytokines in BALF CD4^+ ^T cells obtained from patients and controls. There was a significant increase in the relative expression of IL-2 mRNA in CD4^+ ^T cells from sarcoidosis patients compared with controls (*p = 0.008*; figure 1c). Both patient subgroups showed significantly increased levels of IL-2 mRNA as compared to controls. There was no significant difference in IFN-γ expression in the whole patient group compared to controls, but when we investigated IFN-γ mRNA levels in patient subgroups, a significantly increased IFN-γ level was observed in non-Löfgren's compared to Löfgren's patients (*p = 0.03*; figure 1d). The mRNA level of IFN-γ was also increased in non-Löfgren's patients versus controls *(p = 0.03*; figure 1d).

We detected no difference in IL-13 mRNA transcripts between patients and controls (figure 1e). IL-4 and IL-5 mRNA expression could only be detected in BALF CD4^+ ^T cells of two patients and one control (data not shown).

### Galectin-9 mRNA expression in BALF CD^4+ ^T cells

It has been reported that the interaction between TIM-3 and its ligand, galectin-9, could downregulate Th1 responses [16]. Because of the decreased TIM-3 expression in BALF CD4^+ ^T cells of patients, we also analyzed the levels of galectin-9 in these cells and found that the expression of galectin-9 in BALF CD4^+ ^cells from patients was similar to that in healthy controls (figure 1f), suggesting a normal galectin-9 expression in sarcoidosis. We found no statistically differences in expression of galectin-9 in BALF CD4^+ ^cells between patient subgroups.

### Correlation between TIM expression in BALF cells and BALF cellular parameters

**Figure 2:**
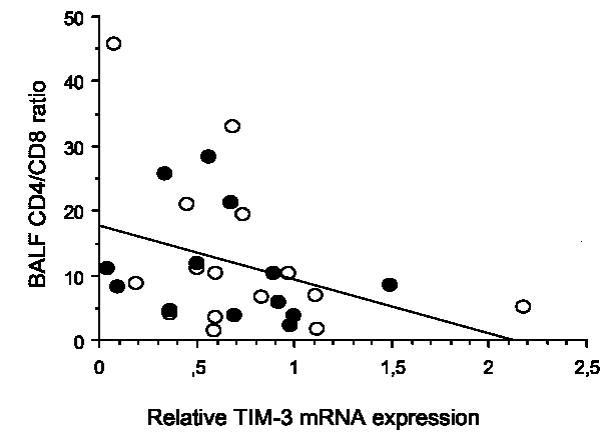
**The levels of TIM-3 mRNA correlated negatively with the ratio of CD4 to CD8 in BALF *(n = 28; r = -0.4, p = 0.035) *in the whole patient group**. The correlation was analysed using Spearman's rank correlation test. Filled circles: Löfgren's patients; open circles: non- Löfgren's patients.

When investigating associations between TIM molecules and BALF cellular parameters in patients, TIM-3 mRNA levels correlated negatively with the ratio of CD4^+ ^to the CD8^+ ^T cells in the lungs of sarcoidosis patients (*r = -0.4, p = 0.035*; figure 2).

### Correlation between TIMs and cytokine expression in BALF cells

**Figure 3:**
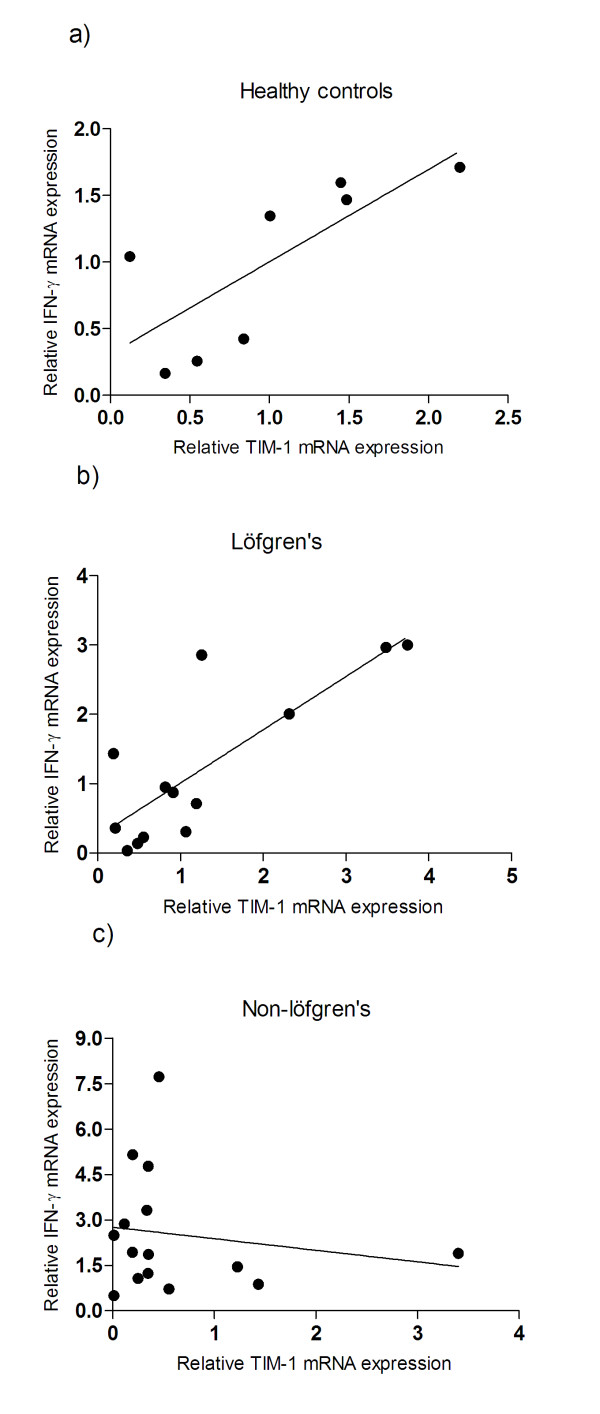
**The mRNA level of TIM-1 correlated with the level of IFN-γ in BALF CD4^+ ^T cells**. The level of TIM-1 and IFN-γ correlated positively in a) healthy controls *(n = 8; r = 0.88, p = 0.02) *and b) Löfgren's patients *(n = 13; r = 0.68, p = 0.01)*, while no correlation was observed in c) non- Löfgren's patients *(n = 15)*. The correlation was analysed using Spearman's rank correlation test.

The level of TIM-1 mRNA correlated positively with the level of IFN-γ in control subjects and in patients with Löfgren's syndrome (*r = 0.83, p = 0.02; r = 0.68, p = 0.01*; respectively, figures 3a, b), while no correlation in non- Löfgren's patients was observed (figure 3c).

### CD^4+^AV2S3^+ ^T cells versus CD4^+^AV2S3^- ^T cells

The mRNA expression of TIM-1, TIM-3, IL-2, IFN-γ and IL-13 was evaluated and compared between isolated CD4^+^AV2S3^+ ^and CD4^+^AV2S3^- ^BALF T cells of patients with lung restricted AV2S3 T cell expansions (n = 12). No differences were detected in mRNA expression for these genes in AV2S3^+ ^versus AV2S3^- ^BALF T cells (data not shown). A tendency to a decreased IL-13 mRNA level in AV2S3^+ ^versus AV2S3^- ^T cells was found, however IL-13 mRNA was detected in cells from only 5 patients.

### Cell surface expression of TIM molecules

**Figure 4:**
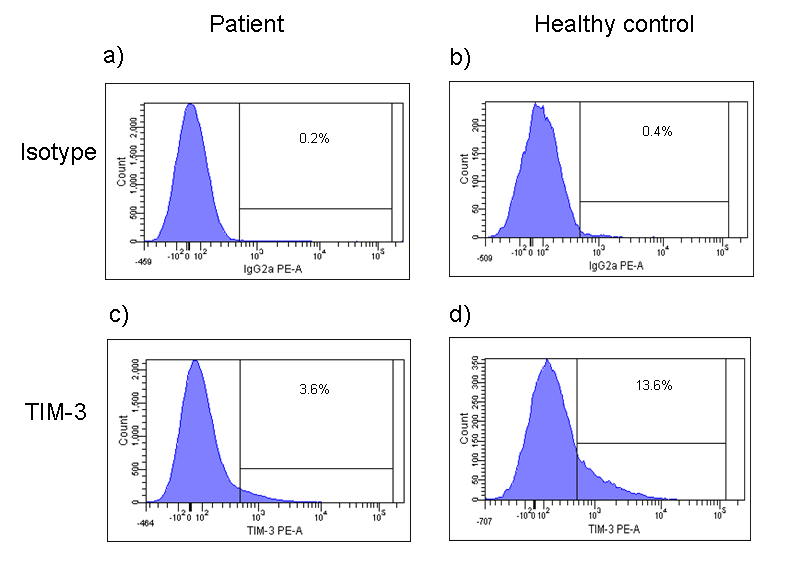
**Flow cytometric analysis of TIM-3 protein expression in CD3^+^CD4^+ ^lymphocytes in bronchoalveolar lavage fluid (BALF)**. The histograms show isotype controls and TIM-3 staining in BALF of one sarcoidosis patient (a, c) and one healthy control (b, d). Figures show representative results from independent experiments.

**Figure 5:**
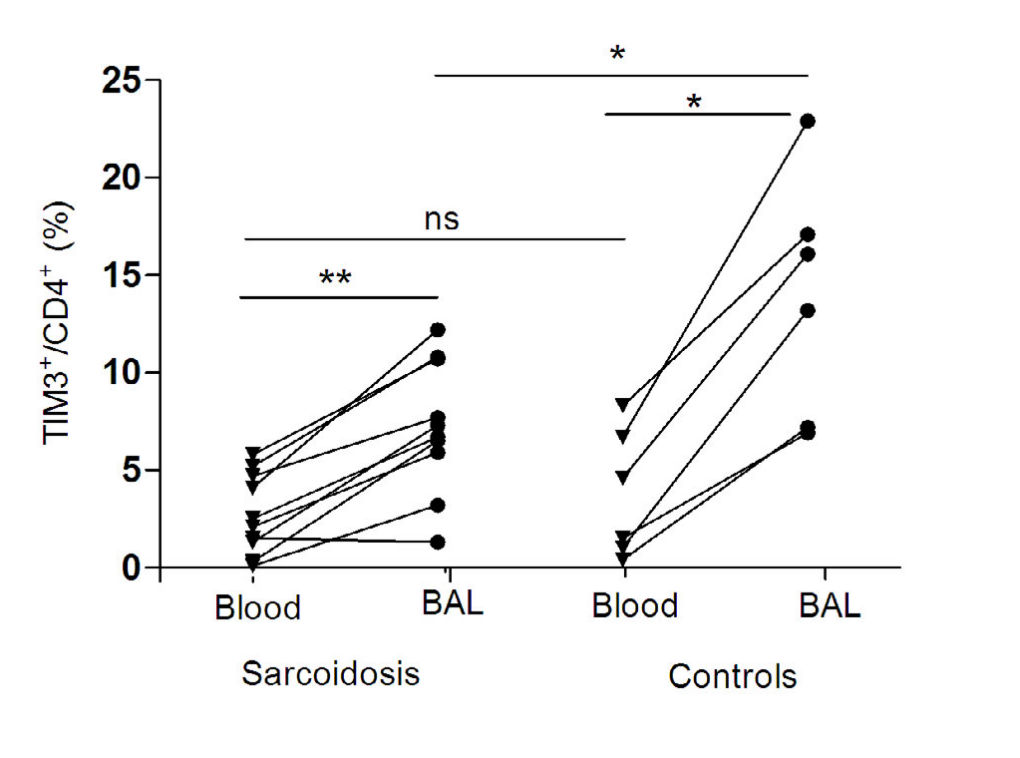
**Flow cytometric analysis of TIM-3 protein expression in CD3^+^CD4^+ ^lymphocytes in bronchoalveolar lavage fluid (BALF) and blood**. TIM-3 cell surface staining on BALF cells showed a lower frequency of TIM-3 expressing CD4^+ ^cells in patients with sarcoidosis (n = 10) versus controls (n = 6) (*p = 0.03*). A higher frequency of TIM-3 expressing cells was observed in BALF versus blood (in patients, p = 0.004; in controls, *p = 0.03)*. The lines indicate T cell subpopulations from the same individual. * p < 0.05, ** p < 0.01.

Using antibodies against human TIM-1 and TIM-3 and flow cytometry, we determined the expression levels of TIM-3 on BALF and blood T cells from patients (n = 10) and controls (n = 6). The relative number of TIM-3 positive BALF CD4^+ ^T cells was significantly reduced in patients compared with controls (*p = 0.03*; figures 4, 5). No difference was observed between patient subgroups. There was no difference in the frequency of TIM-3- expressing blood CD4^+ ^T cells between patients and controls. Both controls and patients had higher percentages of CD4^+ ^cells expressing TIM-3 in BALF versus blood (in controls: *p < 0.05*, in patients: p < 0.01; figure 5).

In addition, analysing the expression of TIM-3 in BALF and blood CD8^+ ^T cells, we found no statistically significant difference either between patients and controls or between patient subgroups (data not shown).

TIM-1 was not detectable on T cells from either patients or controls.

## Discussion

In this study, we demonstrate a decreased mRNA and protein level of TIM-3 in BALF CD4^+ ^T cells of sarcoidosis patients with active disease, while IL-2 mRNA expression was elevated. In comparison to Löfgren's patients, patients without Löfgren's syndrome had reduced TIM-1 mRNA levels, whereas the IFN-γ mRNA level was increased.

The investigation of CD4^+ ^T cell clones from cerebral spinal fluid (CSF) of patients with MS (a Th1-mediated disease) and control subjects showed that TIM-3 expression was down-regulated in MS [25]. Indeed, these clones secreted significantly higher amounts of IFN-γ, but expressed reduced levels of TIM-3 mRNA compared to clones from healthy subjects. By using small inhibitory (si) RNA, to knock down TIM-3 expression, increased T-cell proliferation and IFN-γ secretion was found [25]. Thus, a down-regulation of TIM-3 has been considered as an essential T-cell defect to control inflammation in diseases such as MS [25].

Sarcoidosis is a Th1-mediated disease with accumulated CD4^+ ^T cells in the lungs resulting in an increased BALF CD4^+ ^to CD8^+ ^T cell ratio, which has become a clinically important marker of sarcoidosis [1]. The observed reduction of TIM-3 mRNA levels in BALF CD4^+ ^T cells of patients was associated with an increased CD4^+ ^to CD8^+ ^ratio in the lungs of patients, implying that a reduced TIM-3 expression on CD4^+ ^BALF T cells may lead to more intensive CD4^+ ^T cell accumulation in the lungs. The down-regulation of TIM-3 on CD4^+ ^but not CD8^+ ^T cells, as analysed by flow cytometry, may allow Th1 cells to escape galectin-9- induced cell-death, which is consistent with previous results from our group demonstrating that BALF lymphocytes from sarcoidosis patients displayed a non-apoptotic morphology and seemed to be resistant to apoptosis [26]. The fact that there was no difference in TIM-3 expression between Löfgren's and non- Löfgren's patients is also in line with the almost identical CD4/CD8 ratios in these patient subgroups, suggesting a similar disability of CD4^+ ^T cells to undergo apoptosis in both patient subgroups.

The decreased TIM-3 expression could hypothetically be caused by a genetic defect that leads to lower expression of TIM-3 on lung T cells in sarcoidosis patients. This notion may be supported by the association of genetic polymorphisms in human TIM-3 with susceptibility to the autoimmune disease rheumatoid arthritis (RA) [27]. We found no association between HLA-DRB1*0301, known to correlate with a better prognosis in sarcoidosis, and TIM-3 expression.

The recently identified Th-17 cells, which are highly pathogenic and implied to be involved in the development of various human autoimmune diseases, have also been shown to express low levels of TIM-3 relative to Th1 cells [28]. In addition, analysing the expression of the TIM-3 ligand, galectin-9, in CD4^+ ^cells showed no difference either between patients and controls or between patient subgroups, suggesting TIM-3 as a key factor involved in the uncontrolled Th1 response in the lungs of patients.

Furthermore, in both controls and patients, we found a higher percentage of TIM-3-expressing CD4^+ ^T cells in BALF versus blood, suggesting more pronounced regulatory mechanisms to be executed by TIM-3 in the BALF compartment compared with the blood.

Our patient group includes a large number of patients with Löfgren's syndrome, which is a rather common disease presentation in Sweden, enabling separate studies of this distinct patient group. However, patients with non-Löfgren's phenotype may be more representative for global sarcoidosis patients. In sharp contrast to patients with Löfgren's syndrome, who usually have a very good prognosis with a high rate of spontaneous disease resolution, Swedish non-Löfgren's patients tend to have a non-resolving disease course (own observation). This has also been demonstrated in numerous studies from different countries, reviewed in [1]. As we recently reported, the Th1 response in the lungs of Löfgren's patients is decreased compared to non-Löfgren's patients [23]. In our present study, non-Löfgren's patients had a relatively increased IFN-γ and a reduced TIM-1 mRNA level compared to Löfgren's patients. The differences in TIM-1 expression may be related to differences in Th1/Th2 profiles. Umetsu et al showed that TIM-1 expression is highly increased on newly activated cells, but with time Th1 cells lose its expression, while it is sustained on Th2 cells [13]. Thus, the down-regulated TIM-1 expression in non-Löfgren's patients goes well with the exaggerated Th1 response in these patients as also previously reported [23]. The reason why there is no difference in TIM-3 expression between patient subgroups despite higher IFN-γ in non-Löfgren patients could be that, even if TIM-3 is a marker of Th1 cells, there is not a strict correlation between the total CD4^+ ^cell mRNA expression of IFN-γ and the mRNA expression of TIM-3 and/or frequency of TIM-3 expressing cells.

Similarly, studies on CSF mononuclear cells obtained from patients with MS revealed that higher mRNA expression of TIM-1 associated with clinical remissions and low expression of IFN-γ [15], which could be due to the regulatory role of TIM-1 in inflammation.

In this study we could not find any differences in expression of the Th2 associated cytokine IL-13 between patient subgroups, and IL-4 and IL-5 were not detectable. However, we previously observed an exaggerated Th1 immune response in non- Löfgren's patients versus Löfgren's patients [23]. The possible role of Th2 cells in the lungs of Löfgren's patients is an unexplored area that needs to be further investigated.

The expansion of AV2S3^+ ^T cells in the lungs of HLA-DRB1*0301 positive patients implies that AV2S3^+ ^T cells have been selected by interaction with a specific antigen [29]. AV2S3^+ ^CD4^+ ^lung T cells express activation markers [30] and high numbers of these cells correlate with disease activity and a better prognosis [31], which indicates a protective roll for this particular T cell subset [8]. Our recent observation of reduced expression of regulatory T cell associated molecules, CD25 [30] and FOXP3 [32] in AV2S3^+ ^compared to AV2S3^- ^cells suggests an effector rather than regulatory function for these cells. However, in this study we found no differences in either TIMs or cytokine expression in AV2S3^+ ^BALF T cells. Further investigations are needed to clarify the exact function of these cells in sarcoidosis.

Strong correlations were observed between TIM-1 molecule mRNA expression and IFN-γ mRNA levels in Löfgren's patients and controls, but not in non-Löfgren's patients. This is in line with an imbalanced pulmonary inflammation especially in non-Löfgren's patients.

## Conclusion

**Figure 6:**
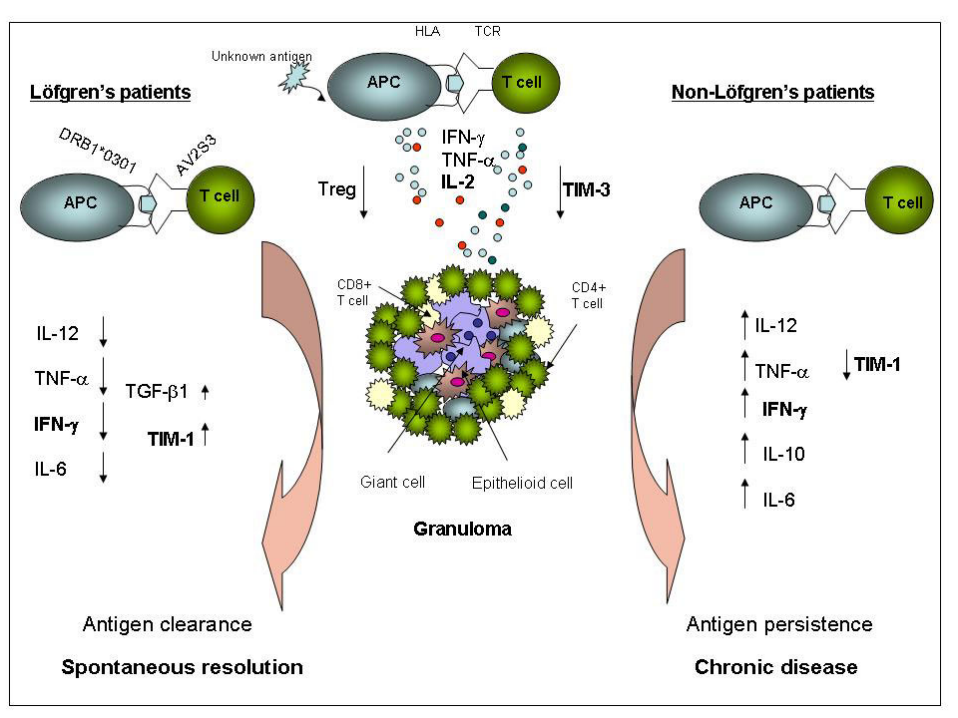
**Hypothetical model of inflammatory reactions in sarcoidosis, comparing the immune response in the lungs of patients with Löfgren's syndrome and those without**. The triggering event is the presentation of an (unknown) antigen by antigen-presenting cells (APC) to T cells. In the lungs of patients, there are fewer regulatory T cells, as well as a lower CD4^+ ^T cell expression of TIM-3, compared to healthy controls. Both these factors may contribute to the characteristic Th1-type inflammation. Differences in the exact type of antigen(s) presented in the respective patient subgroups, as well as antigen clearance or persistence, may contribute to the enhanced Th1 response seen in non-Löfgren's patients, which is associated with a lower CD4^+ ^T cell TIM-1 expression compared to Löfgren's patients. Markers indicated in bold are those included in the present study, for the others see [22,31] and [32].

We demonstrate here a decreased mRNA and protein expression of TIM-3 in the BALF CD4^+ ^T cells of sarcoidosis patients versus controls and decreased mRNA expression of TIM-1 in non-Löfgren's patiens versus Löfgren's patients (figure 6) [33]. Further studies on human TIM-3 polymorphisms and function in sarcoidosis may provide us with knowledge to further explain the pathogenesis of the disease. The reduced TIM-1 expression together with increased IFN-γ levels in non-Löfgren's patients may relate to the more pronounced inflammatory reaction in these patients. Collectively, the reduced TIM-3 expression on Th1 cells in inflammatory sites may represent a T cell defect to control the Th1 response, which might contribute to the accumulation of inflammatory cells in the lungs and the pathogenesis of sarcoidosis.

## Competing interests

The authors declare that they have no competing interests.

## Authors' contributions

FI participated in the design of the study and performed the practical lab works, the statistical analysis and wrote the manuscript. JW participated in the design of study and helped to draft the manuscript. BD helped in the flow cytometry analysis. MK and TO carried out the design and optimising of TIMs probes and primers. AE participated in the design of study and helped to draft the manuscript. JG conceived the study, and participated in its design and coordination and helped to draft the manuscript. All authors have read and approved the final manuscript.
